# Evaluation and Pre-selection of New Grapevine Genotypes Resistant to Downy and Powdery Mildew, Obtained by Cross-Breeding Programs in Spain

**DOI:** 10.3389/fpls.2021.674510

**Published:** 2021-12-10

**Authors:** Leonor Ruiz-García, Pilar Gago, Celia Martínez-Mora, José Luis Santiago, Diego J. Fernádez-López, María del Carmen Martínez, Susana Boso

**Affiliations:** ^1^Department of Biotechnology, Genomics and Plant Breeding, Instituto Murciano de Investigación y Desarrollo Agrario y Alimentario, Murcia, Spain; ^2^Department of Viticulture and Forestry, Misión Biológica de Galicia (Consejo Superior de Investigaciones Científicas, CSIC), Salcedo, Spain

**Keywords:** downy mildew, powdery mildew, resistance, grapevine, marker-assisted breeding, *Vitis*

## Abstract

The need to develop an environmentally friendly, sustainable viticulture model has led to numerous grapevine improvement programmes aiming to increase resistance to downy and powdery mildew. The success of such programmes relies on the availability of protocols that can quantify the resistance/susceptibility of new genotypes, and on the existence of molecular markers of resistance loci that can aid in the selection process. The present work assesses the degree of phenotypic resistance/susceptibility to downy and powdery mildew of 28 new genotypes obtained from crosses between “Monastrell” and “Regent.” Three genotypes showed strong combined resistance, making them good candidates for future crosses with other sources of resistance to these diseases (pyramiding). In general, laboratory and glasshouse assessments of resistance at the phenotype level agreed with the resistance expected from the presence of resistance-associated alleles of simple sequence repeat (SSR) markers for the loci *Rpv3* and *Ren3* (inherited from “Regent”), confirming their usefulness as indicators of likely resistance to downy and powdery mildew, respectively, particularly so for downy mildew.

## Introduction

Downy and powdery mildew (caused by *Plasmopara viticola* and *Erysiphe necator*, respectively) are some of the most important diseases of grapevines worldwide. Both reduce crop yield and quality leading to economic losses. *Vitis vinifera* is highly susceptible to both, while American and Asian vine species are much more resistant, perhaps due to their co-evolution with the causal pathogens (Armijo et al., 2016). The most efficient way to deal with them is chemical control, but this can have a negative biological and ecological impact; sustainable and environmentally friendly viticulture requires such treatment be reduced. Since the discovery of sources of resistance to these pathogens, many grapevine genetic improvement programmes around the world have tried to produce quality grapevine hybrids carrying the resistance genes of wild American vine species - but this has not been easy (Töpfer et al., 2011; Villano and Aversano, 2020). We still lack knowledge on the molecular bases of such resistance, and several backcrosses are often required before a hybrid of sufficient winemaking quality and lasting disease resistance is obtained. Such programmes have involved the use of American vine species, e.g., *Vitis riparia*, *Vitis rupestris*, *Muscadinia rotundifolia*, *Vitis cinerea*, etc., or species from the Far East, such as *Vitis piasezkii*, *Vitis amurensis*, *Vitis romanetii*, or *Vitis vinifera* Kishmish vatkana (Merdinoglu et al., 2018; Maul and Töpfer, 2019). These show either partial resistance, or, in the case of *M. rotundifolia* and *V. piasezkii*, complete resistance (Wan et al., 2007; Bellin et al., 2009; Casagrande et al., 2011; Gessler et al., 2011). Many studies have also tried to compare the degree of resistance to *P. viticola* and *E. necator* of the hybrids produced in different improvement programmes (Hoffmann et al., 2008; Kozma et al., 2009; Vezzulli et al., 2017, 2019; Bove et al., 2020; Possamai et al., 2020).

To date, 31 grapevine genomic regions have been associated with resistance to downy mildew (*Rpv* loci) and 13 with resistance to powdery mildew (*Run* loci and *Ren* loci) according to the table of loci for traits in grapevine relevant for breeding and genetics (update April 30, 2021^1^). The availability of markers that reveal the presence of these loci could allow for the marker-assisted selection (MAS) of likely resistant genotypes (Eibach et al., 2007; Kozma et al., 2009; Vezzulli et al., 2019; Zini et al., 2019). The resistance actually shown by a genotype can then be tested in the field and under controlled laboratory/glasshouse conditions following established protocols (Brown et al., 1999; Boso et al., 2006, 2010, 2014; Prajongjai et al., 2014). New material resistant to both diseases coming out of improvement programmes includes the variety “Regent.” Obtained at the Julius-Kühn Institute in Germany, its pedigree includes American vines carrying *Ren3*, *Ren9*, *Rpv3*, *Rpv4*, and *Rpv11* (Fischer et al., 2004; Welter et al., 2007; Zendler et al., 2017).

The aim of the present work was to assess the degree of phenotypic resistance/susceptibility to downy and powdery mildew of 28 “Monastrell” × “Regent” hybrids selected for their carriage of resistance-associated alleles of simple sequence repeat (SSR) markers for the loci *Rpv3* and *Ren3*, and thus being likely resistant to these diseases. The results allow for the selection of genotypes that could be used in future crosses with other sources of resistance to downy and powdery mildew (pyramiding).

## Materials and Methods

### Plant Material

The study material included 28 new genotypes obtained by crosses in 2012 (Ruiz-García et al., 2014) between “Monastrell” (susceptible to downy mildew and with medium resistance to powdery mildew) and “Regent” which carries the loci *Rpv3* and *Ren3* (Fischer et al., 2004; Welter et al., 2007).

The identity of the parentals and of the 28 genotypes produced from them was performed by PCR amplification of nine SSR markers registered in the NCBI database^2^ (Supplementary Table 1).

### Amplification of Resistance-Associated Molecular Markers of *Rpv3* and *Ren3*

The resistance-associated molecular markers of *Rpv3* and *Ren3* used in this work and their sequences are shown in Supplementary Table 2. PCR analyses to detect the presence of the SSR alleles-associated to resistance were performed according to Bayo-Canha et al. (2019). “Monastrell” and “Regent” were used as negative and positive controls, respectively.

### Resistance to Downy and Powdery Mildew

Resistance to downy mildew was examined using the leaf disc test (laboratory conditions) (Rumbolz et al., 2002). Susceptibility to powdery mildew was examined using the (modified) method of Wang et al. (1995) (glasshouse conditions). Resistance to both diseases was also recorded *via* the use of official OIV descriptors (Organisation Internationale de la Vigne et du Vin [OIV], 2009), with small modifications. All tests were performed in triplicate. “Monastrell” and “Regent” were used as controls for the resistance assays.

#### Plant Material Used

At least 60 cuttings with 2–3 buds were taken in January from each of the field-grown genotypes. These were disinfected, dipped in paraffin wax and preserved in a cold chamber for at least 8 weeks to encourage later sprouting in a glasshouse under controlled conditions of temperature and humidity (25°C, RH > 95%, 16 h white light at 400–700 nm and 8 h darkness). Thirty plants of each genotype were used to examine resistance to either disease.

#### Pathogen Material

*Plasmopara viticola* and *Erysiphe necator* were obtained from plants naturally infected in the experimental vineyards of the *Misión Biológica de Galicia* (MBG-CSIC). For *P. viticola*, sporangia for inocula were propagated following the method of Rumbolz et al. (2002). For *E. necator*, no propagation was needed since plenty of conidia were already available on infected ‘Castañal’ host plants. To prepare the inoculum, fresh conidia were collected using a small paintbrush, placed in centrifuge tubes, and diluted with 50 ml of distilled water with 0.05% Tween 20, thus obtaining a final concentration of 50,000 conidia ml^–1^.

##### Resistance to Downy Mildew (Laboratory Leaf Disc Tests)

The 5th or 6th unfurled leaf on a green shoot of each of the 30 plants per genotype grown in the glasshouse (see above) was detached. Leaf discs were prepared and incubated according to Rumbolz et al. (2002). Disease incidence and disease severity were visually analysed as independent variables at 5 days post-inoculation (dpi) according to the method of Boso et al. (2014).

##### Resistance to Powdery Mildew (Glasshouse Conditions)

Ten plants per genotype were challenged on their adaxial leaf surfaces by spraying with the prepared conidial suspension. They were then incubated for 5–6 days at 24°C, at an RH of <55%, and under long day conditions (white light at 400–700 nm, 16 h light and 8 h dark). At 5–6 dpi, disease incidence was calculated as the number of leaves with sporulating lesions per total number of leaves per plant, and disease severity as the percentage of leaf area showing symptoms of sporulation.

##### Disease Assessment Using Organisation Internationale de la Vigne et du Vin Descriptors

Descriptors recommended by the OIV [OIV452-1 for downy mildew (leaf discs inoculated with *P. viticola* sporangia) and OIV455-1 for powdery mildew (leaf in glasshouse inoculated with *E. necator*)] (Organisation Internationale de la Vigne et du Vin [OIV], 2009). Different scores for resistance to downy and powdery mildew are available in Supplementary Figures 1, 2.

### Statistical Analysis

The differences between the studied variables were analysed by ANOVA using the fixed effects model (*p* < 0.001). Following ANOVA, significant *F* values were subjected to comparison using Fisher’s protected least significant difference (LSD) test (*p* < 0.05). The association between phenotypic resistance and that expected from the possession of resistance-associated alleles of the SSR markers was determined *via* the Chi squared test. All calculations were made using SAS V8.1 software (SAS Institute, Cary, NC; 2000).

## Results

### Presence of Resistance-Associated Simple Sequence Repeat Alleles

#### Rpv3

Of the 28 genotypes examined, 18 were positive for the resistance-associated SSR allele UDV305_**299** bp, 18 for UDV737_**279** bp, 18 for UDV108_**238** bp, and 18 for GF18-8_**392** bp (Supplementary Table 3). The 18 genotypes that carried all four alleles were deemed likely resistant to downy mildew.

#### Ren3

Of the 28 genotypes examined, 20 were positive for the resistance-associated SSR allele GF15-42_**199** bp, 19 for GF15-28_**341** bp, 21 for GF15-30_**446** bp, and 21 for VChr15CenGen06_**283** bp (Supplementary Table 3). The 19 genotypes that carried all these alleles were deemed likely resistant to powdery mildew.

Nine genotypes (genotypes 4_136, 5_022, 5_033, 5_107, 6_018, 6_025, 6_046, 6_080, and 6_125) carried the resistance-associated SSR alleles for both *Rpv3* and *Ren3* (Supplementary Table 3), and were deemed likely resistant to both diseases.

### Phenotypic Resistance

#### Downy Mildew

**FIGURE 1:**
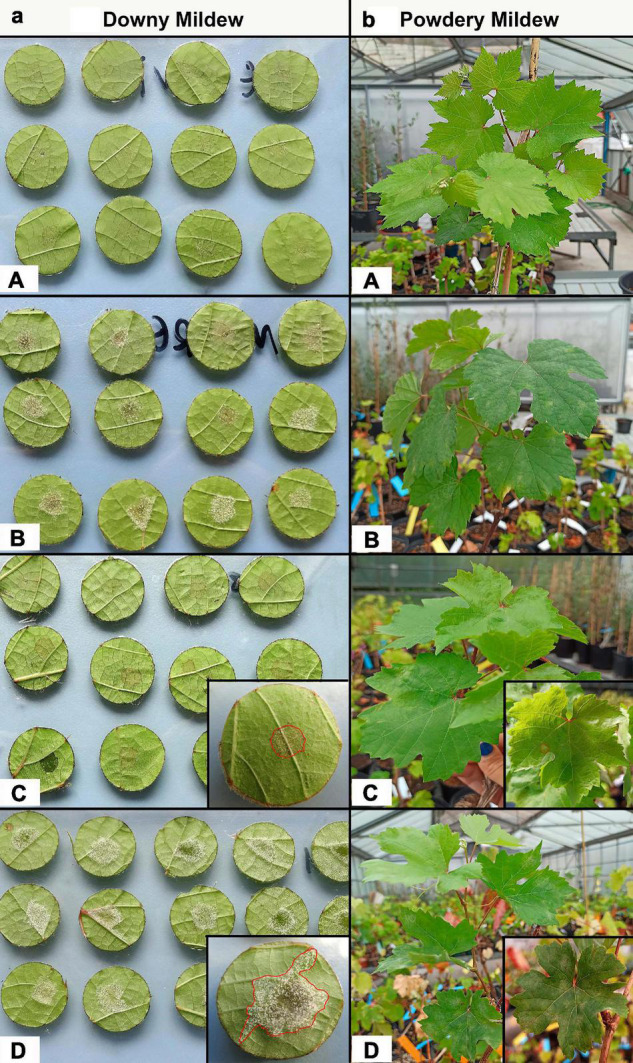
**(a)** Images of leaf discs from genotypes showing different degrees of resistance to downy mildew (6 dpi), as well as greater resistance than “Regent” (no sporulation) or greater susceptibility than “Monastrell” (white downy mildew sporulation on the abaxial surface of leaf discs). **(A)** “Regent”; **(B)** “Monastrell”; **(C)** genotype 6_080 (resistant); **(D)** genotype 4_001 (susceptible). **(b)** Images of plants, grown under glasshouse conditions, belonging to genotypes showing different degrees of resistance to powdery mildew (6 dpi). **(A)** “Regent”; **(B)** “Monastrell”; **(C)** genotype 5_107 (resistant); **(D)** 5_060 (susceptible).

“Monastrell” showed high disease incidence (100%) and severity scores (58%), while “Regent” showed medium incidence (54%) and low severity (8%) scores. With respect to descriptor code OIV 452-1, “Monastrell” showed a score of 1, and “Regent” a score of 5 (Table 1). Among the 27 new genotypes tested, disease incidence ranged between 9 and 100%, and disease severity between 5 and 67%. With respect to the same descriptor code, and taking incidence and severity into account, six genotypes showed a resistance score of 9, six a score of 7, four a score of 5, one a score of 3, and 10 a score of 1. It should be noted that 12 genotypes (3_032, 3_082, 4_032, 4_063, 4_082, 5_022, 5_033, 5_060, 5_078, 5_107, 6_046, and 6_080) were transgressive with respect to the resistance donor “Regent” (OIV = 5), and showed greater resistance (OIV = 9 and 7). “Monastrell” and genotypes 3_094, 4_005, 4_037, 4_011, 4_136, 3_070, 3_073, 4_001, and 3_052 were the least resistant of all; they showed significantly higher incidence and severity scores (Table 1 and Supplementary Figure 3). Genotype 6_080 was significantly more resistant than the rest, with low severity and incidence scores (Figure 1a). Genotypes 5_078, 4_082, 4_063, 5_022, and 5_107 showed somewhat less resistance, with slightly higher incidence values but similar severity values. The remaining genotypes showed medium resistance, with intermediate disease incidence and severity scores.

**TABLE 1:** Mean disease severity (DS), disease incidence (DI), OIV scores, and resistance genotype for downy mildew and powdery mildew.

	Phenotype post-inoculation with downy mildew					Phenotype post-inoculation with powdery mildew					
			
Vine material	DS (%)	S.D.	DI (%)	S.D.	OIV (452-1)	DS (%)	S.D.	DI (%)	S.D.	OIV (455-1)	*Genotype
Monastrell	58.3*^a^*	14.43	100.0a	0.00	1	65.0a	13.23	49.3ab	4.04	5	Susceptible
Regent	8.3cd	2.89	54.0bcd	2.00	5	8.3c	2.89	25.0de	0.00	9	DM_PM
3_016	5.0d	0.00	52.0bcde	0.00	5	0.0d	0.00	2.3ij	2.52	9	DM
3_025	not data	not data	not data	not data	not data	25.0b	0.00	13.6fgh	321%	9	DM
3_032	5.0d	0.00	36.3fghi	3.51	7	5.0cd	0.00	21.6ef	5.77	9	DM
3_052	46.6ab	20.21	100.0a	0.00	1	5.0cd	0.00	5.0hij	0.00	9	PM
3_058	30.0bc	5.00	100.0a	0.00	1	0.0d	0.00	0.0j	0.00	9	PM
3_070	50.0ab	25.00	100.0a	0.00	1	5.0cd	0.00	12.6fghi	2.52	9	PM
3_073	48.3ab	37.86	100.0a	0.00	1	5.0cd	0.00	1.6j	2.89	9	PM
3_082	5.0d	0.00	45.6cdef	2.08	7	25.0b	0.00	45.0ab	18.03	5	DM
3_094	63.3a	12.58	100.0a	0.00	1	5.0cd	0.00	0.0j	0.00	9	PM
4_001	48.3ab	37.86	100.0a	0.00	1	25.0b	0.00	33.3cd	14.43	7	PM
4_005	66.67a	14.43	100.0a	0.00	1	5.0cd	0.00	0.0j	0.00	9	PM
4_011	50.0ab	25.00	100.0a	0.00	1	5.0cd	0.00	1.6j	2.89	9	PM
4_032	11.6cd	11.55	40.0defg	0.00	7	5.0cd	0.00	28.0de	5.20	7	DM
4_037	55.0a	8.66	100.0a	0.00	1	5.0cd	0.00	9.3ghij	4.04	9	PM
4_063	5.0d	0.00	23.3ij	9.87	9	25.0b	0.00	40.0bc	15.00	5	DM
4_082	5.0d	0.00	20.0jk	0.00	9	5.0cd	0.00	6.6ghij	2.89	9	DM
4_124	11.6cd	11.55	65.0b	17.32	3	25.0b	0.00	40.0bc	15.00	5	DM
4_136	50.0ab	25.00	100.0a	0.00	1	5.0cd	0.00	17.3efg	5.86	9	DM_PM
5_022	18.3cd	11.55	25.6hij	18.01	9	5.0cd	0.00	1.6j	2.89	9	DM_PM
5_033	5.0d	0.00	44.3cdef	12.01	7	5.0cd	0.00	23.3def	7.64	9	DM_PM
5_060	5.0d	0.00	38.9efgh	10.65	7	65.0a	13.23	53.3a*	5.77	3	DM
5_078	5.0d	0.00	20.0jk	4.00	9	5.0cd	0.00	10.0ghij	0.00	9	PM
5_107	5.0d	0.00	30.0ghij	0.00	9	1.6d	0.00	0.0j	0.00	9	DM_PM
6_018	5.0d	0.00	55.6bc	25.58	5	5.0cd	0.00	21.6ef	2.89	9	DM_PM
6_025	5.0d	0.00	55.3bc	21.57	5	5.0cd	0.00	7.5ghij	3.54	9	DM_PM
6_046	11.6cd	11.55	40.3defg	4.51	7	5.0cd	0.00	6.6ghij	2.89	9	DM_PM
6_080	5.0d	0.00	8.6k	4.16	9	5.0cd	0.00	3.0hij	1.73	9	DM_PM
6_125	11.0cd	12.17	51.3bcde	1.15	5	5.0cd	0.00	1.6j	2.89	9	DM_PM

**LSD (0.05)**	24.49		14.15			5.71		2.95			

*S.D., standard deviation. ^a^Means with the same letter are not significantly different (LSD test).*

*Result for resistance based on the molecular findings: PM, Powdery mildew resistant; DM, Downy mildew resistant; PM_DM, Powdery and downy midew resistant.

#### Powdery Mildew

“Monastrell” showed high incidence (50%) and severity (65%) scores, while “Regent” showed scores of only 25 and 8%, respectively (*p* < 0.01). With respect to descriptor code OIV 455-1, “Monastrell” had a score of 5, while “Regent” scored 9. For the 28 new genotypes tested, disease incidence ranged between 0 and 53%, while severity ranged from 0 to 65%. With respect to the same descriptor code, and taking incidence and severity into account, 22 genotypes showed a resistance score of 9, two a score of 7, three a score of 5, and one a score of 3 (Table 1). With respect to descriptor code OIV 455-1, genotype 5_060 showed transgressive segregation and less strong resistance (OIV = 3) than “Monastrell” (OIV = 5). Genotype 5_060 and “Monastrell” showed significantly higher incidence and severity scores compared to the rest (Table 1; Figure 1b; Supplementary Figure 3). Genotypes 3_082, 4_063 and 4_124 showed medium resistance (lower severity score). “Regent” grouped with the genotypes showing the lowest incidence and severity values (Table 1 and Supplementary Figure 3); genotypes 3_058 and 5_107 showed no symptoms of disease at all (Table 1).

#### Combined Resistance to Both Diseases

Genotypes 6_080, 5_107 and 4_082 showed the greatest combined resistance to both diseases, with incidence and severity values much lower than those shown even by “Regent” (Table 1).

### Association Between Phenotypic Resistance and Possession of Resistance-Associated Simple Sequence Repeat Alleles

#### Rpv3

Supplementary Table 4 shows the association between possession of the SSR alleles UDV305_**299** bp, UDV737_**279** bp, UDV108_**238** bp, and GF18-8_**392** bp and the actual phenotypic resistance shown to downy mildew. Of the 16 genotypes (3_016, 3_032, 3_082, 4_032, 4_063, 4_082, 5_022, 5_033, 5_060, 5_078, 5_107, 6_018, 6_025, 6_046, 6_080, and 6_125) with resistance equal to or greater than that shown by “Regent” (OIV 452-1 = 9, 7, and 5), 15 (all genotypes except for 5_078) possessed all four alleles (Supplementary Table 3). Genotype 5_078 gave a false negative result for resistance based on the molecular findings (Table 1). Of the 11 genotypes (3_052, 3_058, 3_070, 3_073, 3_082, 3_094, 4_001, 4_005, 4_011, 4_032, 4_037, 4_063, 4_082, 4_124, and 4_136) showing less resistance than “Regent” (OIV 452-1 = 1 and 3), nine (all genotypes except for 4_124 and 4_136) did not have all the above alleles (Supplementary Table 3). Genotypes 4_124 and 4_136 gave false positive results for resistance based on molecular findings (Table 1). The carriage of all four alleles was significantly associated (χ^2^ = 19.57, *p* ≤ 0.001) with actual phenotypic resistance.

#### Ren3

Supplementary Table 4 also shows the association between detection of the SSR alleles GF15-42_**199** bp, GF15-28_**341** bp, GF15-30_**446** bp, and VChr15CenGen06_**283** bp and actual phenotypic resistance shown to powdery mildew. Of the 24 genotypes (3_016, 3_025, 3_032, 3_052, 3_058, 3_070, 3_073, 3_094, 4_001, 4_005, 4_011, 4_032, 4_037, 4_082, 4_136, 5_022, 5_033, 5_078, 5_107, 6_018, 6_025, 6_046, 6_080, and 6_125) with resistance similar to “Regent” (OIV 455-1 = 7 and 9), 19 (all genotypes except 3_016, 3_025, 3_032, 4_032 and 4_082) carried all four alleles (Supplementary Table 3). Genotypes 3_016; 3_025; 3_032; 4_032; 4_082 gave false negative results based on molecular findings (Table 1). The four genotypes (3_082, 4_063, 4_124 and 5_060) that showed actual phenotypic resistance well below that shown by “Regent” (OIV 455-1 = 1, 3 or 5) did not carry all four alleles (Supplementary Table 3). The carriage of all four alleles was significantly associated (χ^2^ = 10.70, *p* ≤ 0.025) with actual phenotypic resistance.

## Discussion

The present leaf disc and plant inoculation results for resistance are reminiscent of those reported by other authors (Staudt and Kassemeyer, 1995; Prajongjai et al., 2014; Vezzulli et al., 2017) who indicate that non-*vinifera* hybrids are not fully resistant to downy and powdery mildew, and that the degree of resistance is a segregable trait. Indeed, in the present work, different degrees of resistance/susceptibility were seen among those genotypes generally classified as resistant or susceptible (Table 1). The greatest resistance to downy mildew was shown by genotypes 6_080 and 4_082, while 4_005 showed the greatest susceptibility. With respect to powdery mildew, genotypes 3_058 and 5_107 showed the greatest resistance, while 5_060 showed the greatest susceptibility. The present data support the idea that transgressive segregation is common in plant breeding populations, with a number of recombinants appearing as outliers with respect to the resistance shown by the parental phenotypes (Mackay et al., 2021). With regard to downy mildew descriptor code OIV 452-1, 12 genotypes were transgressive with respect to the resistance donor “Regent” (OIV = 5), showing greater resistance than that genotype (OIV = 7 or 9). Similar results were obtained by Vezzulli et al. (2019) in a segregating population for resistance to downy mildew. These extreme phenotypes suggest the presence of unidentified resistance factors that segregate in the breeding populations and result in minor but significant effects. The causes of transgressive segregation may be genetic (positive or negative complementation of additive alleles, epistatic interactions of unique parental attributes, the unmasking of recessive alleles from a heterozygous parent, or any combinations of these mechanisms) or environmental.

Zanghelini et al. (2019) agree with the present hyopthesis that a more environmentally friendly way to control grapevine disease would be to select new genotypes with combinations of resistance loci. In the present work, genotypes 6_080, 5_107 and 4_082 showed the greatest resistance to both downy and powdery mildew, and indeed these genotypes have been selected as the best progenitors in an IMIDA breeding program with the aim of combining their characteristics with those conferred by other resistance loci to downy mildew (e.g., *Rpv10*) and powdery mildew (e.g., *Ren1*), and thus help maintain the durability of resistance (pyramiding) (Eibach et al., 2007).

The possession of alleles of the SSRs used as markers of resistance was significantly associated with the actual phenotypic resistance to downy (especially) and powdery mildew, confirming that these markers can be used in plant improvement programmes designed to reduce the current use of pesticides. With respect to downy mildew, only two false positives were obtained (by genotypes 4_124 and 4_136, i.e., they were supposedly resistant but actually susceptible), and one false negative (by genotype 5_078, i.e., supposedly susceptible but actually showed resistance). With respect to powdery mildew, five false negative results were noted (provided by genotypes 3_016, 3_025, 3_032, 4_032, and 4_082. The additional fine mapping of the areas of the genome where different *R*-*loci* have been identified might provide more robust markers for use in marker-assisted selection (Zini et al., 2019; Zendler et al., 2021). Overall, these results confirm that MAS can be of great use in traditional improvement programmes, allowing for the selection of material with resistance to disease. However, MAS is not always as efficient as expected, possibly due to a relatively loose association between QTL alleles and the level of infection (Hospital, 2009).

The introduction of resistance genes from *Vitis* species into a *V. vinifera* variety is a long and costly process, and in any event resistance may be overcome by particularly virulent pathogens. This is why the durability of resistance is crucial - particularly in the case of a perennial species like the vine. Plants that combine several resistance factors might be expected to show greater durability of resistance, even if they display the same level of resistance as those bearing only one resistance factor. MAS can be used to identify those genotypes that combine desired resistance factors, thus helping in the generation of varieties of greater potential for resistance durability. However, it does not appear to be so useful in identifying small-effect loci that can enhance the protection conferred by major genes and thus improve their durability (Merdinoglu et al., 2018). Hence the importance of combining MAS with phenotypic characterisation; this should allow for the better determination of the degree of resistance. Breeding programs should be vigilant of any advances made in molecular biology and genomic selection that might help construct varieties with highly durable resistance (Meuwissen et al., 2001; Merdinoglu et al., 2018).

In conclusion, cross-breeding programs generate great variation and allow for the selection of new genotypes that can promote a more sustainable and environmentally friendly form of viticulture - as long as the winemaking quality of their grapes is confirmed. This variation may include extreme phenotypes that show greater resistance than the actual donor. The combination of phenotypic characterisation and molecular selection is very useful, allowing the degree of resistance achieved in new genotypes, and the durability of that resistance, to be more accurately determined. The very resistant lines discussed in the present work provide valuable material for obtaining durably resistant genotypes, and should help characterise the molecular basis of resistance to downy and powdery mildew.

## Data Availability Statement

The original contributions presented in the study are included in the article/Supplementary Material, further inquiries can be directed to the corresponding author.

## Author Contributions

LR-G, SB, and MM proposed the study, planned and directed it, set goals, undertook experimental work, analysed and interpreted the results, and wrote the draft of the manuscript. JS helped to wrote the draft of the manuscript. PG and CM-M undertook experimental work and helped to wrote the draft of the manuscript. DF-L undertook experimental work. All authors contributed to the final version and read and approved the final manuscript.

## Conflict of Interest

The authors declare that the research was conducted in the absence of any commercial or financial relationships that could be construed as a potential conflict of interest.

## Publisher’s Note

All claims expressed in this article are solely those of the authors and do not necessarily represent those of their affiliated organizations, or those of the publisher, the editors and the reviewers. Any product that may be evaluated in this article, or claim that may be made by its manufacturer, is not guaranteed or endorsed by the publisher.

## Footnotes

1
https://www.vivc.de/

2
https://www.ncbi.nlm.nih.gov/

## Supplementary Material

Supplementary Figure 1 Resistance to downy mildew as recorded by OIV 452-1 descriptors (leaf discs inoculated with *P. viticola* sporangia). The scores for resistance to downy mildew were: OIV-1, very low resistance (dense sporulation over large lesions, 100% of discs affected); OIV-3, low resistance (dense sporulation over medium-sized lesions, 65–99% of discs affected; OIV-5, medium resistance (little sporulation over small-mid size lesions, 47–64% of discs affected; OIV-7, strong resistance (scant sporulation over small lesions; 31–46% of discs affected); OIV-9, very strong resistance (scant sporulation over small lesions; <30% of discs affected).

Supplementary Figure 2 Resistance to powdery mildew as recorded by descriptor OIV 455-1 (leaf in glasshouse inoculated with *E. necator*). The scores for resistance to powdery mildew were: 1, very low resistance [dense sporulation over the entire leaf surface (Adaxial) (estimating an average for all leaves)], >75 of plants affected); 3, low resistance (dense sporulation over 65–100%, 50–75% of plants affected); 5, medium resistance (sporulation over 25–65%, 36–50% of plants affected); 7, strong resistance (scant sporulation over 5–25, 25–35% of plants affected); 9, very strong resistance (sporulation over ≤5%, <25% plants affected).

Supplementary Figure 3 Relationship between disease incidence and severity for downy (leaf disc test) and powdery (glasshouse observation) mildew. Alleles of DMR = SSR alleles associated with resistance to downy mildew (all four alleles present). Alleles of PMR = SSR alleles associated with resistance to downy mildew (all four alleles present). DM_PM resistant to downy and powdery mildew.
