# Supplementary figures and images for: Evaluation and Pre-selection of New Grapevine Genotypes Resistant to Downy and Powdery Mildew, Obtained by Cross-Breeding Programs in Spain
Source: Front Plant Sci. 2021 Dec 10;12:674510. doi: 10.3389/fpls.2021.674510 (PMC8703198; doi:10.3389/fpls.2021.674510)

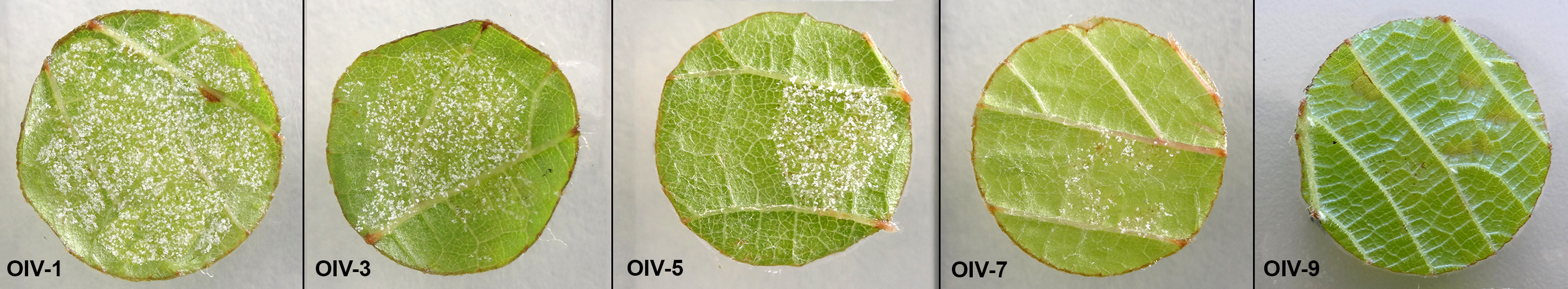

Supplement: Supplementary Figure 1 — Resistance to downy mildew as recorded by OIV 452-1 descriptors (leaf discs inoculated with P. viticola sporangia). The scores for resistance to downy mildew were: OIV-1, very low resistance (dense sporulation over large lesions, 100% of discs affected); OIV-3, low resistance (dense sporulation over medium-sized lesions, 65–99% of discs affected; OIV-5, medium resistance (little sporulation over small-mid size lesions, 47–64% of discs affected; OIV-7, strong resistance (scant sporulation over small lesions; 31–46% of discs affected); OIV-9, very strong resistance (scant sporulation over small lesions; <30% of discs affected). [file Image_1.jpg]

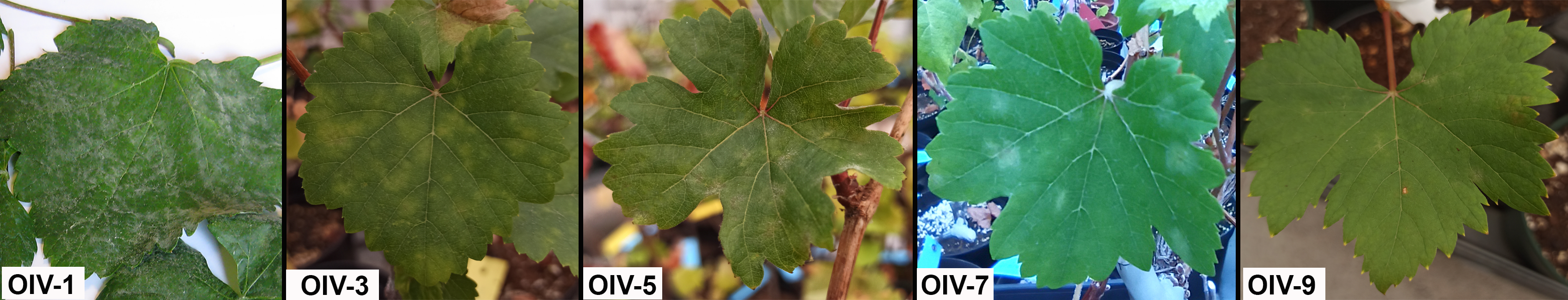

Supplement: Supplementary Figure 2 — Resistance to powdery mildew as recorded by descriptor OIV 455-1 (leaf in glasshouse inoculated with E. necator). The scores for resistance to powdery mildew were: 1, very low resistance [dense sporulation over the entire leaf surface (Adaxial) (estimating an average for all leaves)], >75 of plants affected); 3, low resistance (dense sporulation over 65–100%, 50–75% of plants affected); 5, medium resistance (sporulation over 25–65%, 36–50% of plants affected); 7, strong resistance (scant sporulation over 5–25, 25–35% of plants affected); 9, very strong resistance (sporulation over ≤5%, <25% plants affected). [file Image_2.jpg]

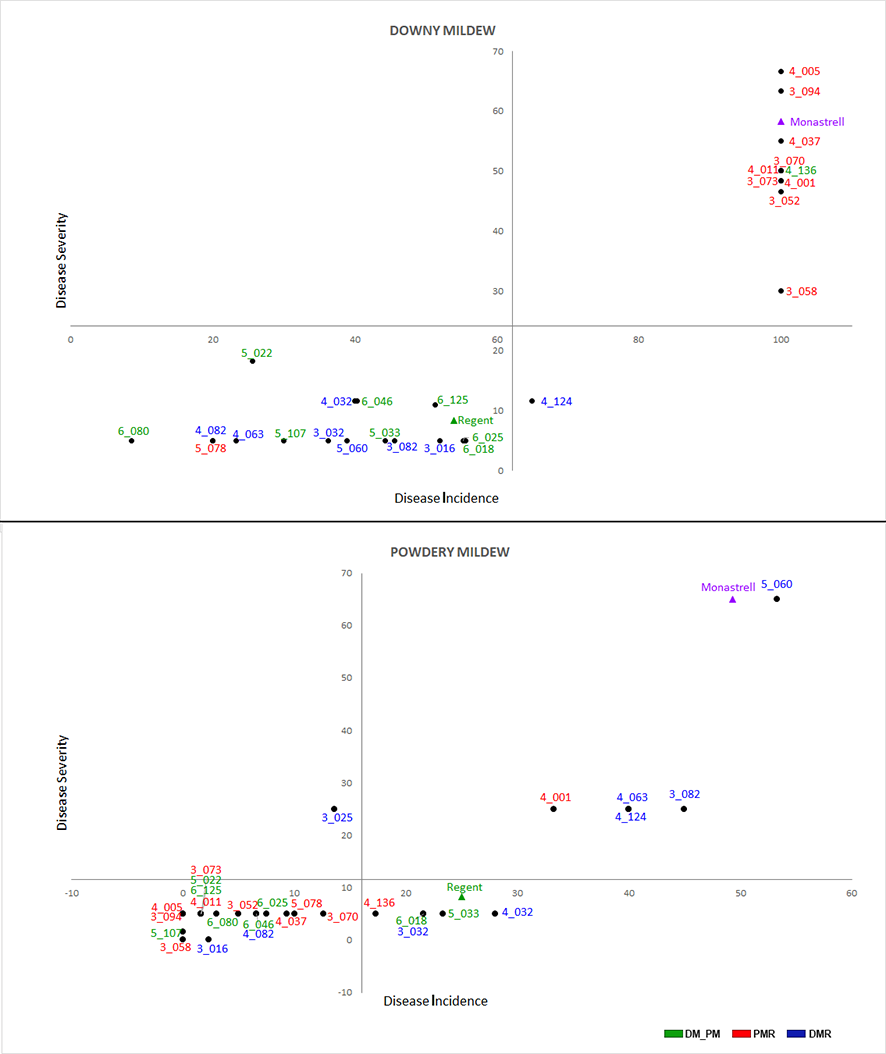

Supplement: Supplementary Figure 3 — Relationship between disease incidence and severity for downy (leaf disc test) and powdery (glasshouse observation) mildew. Alleles of DMR = SSR alleles associated with resistance to downy mildew (all four alleles present). Alleles of PMR = SSR alleles associated with resistance to downy mildew (all four alleles present). DM_PM resistant to downy and powdery mildew. [file Image_3.TIF]
