# Supplementary material for: Evaluation and Pre-selection of New Grapevine Genotypes Resistant to Downy and Powdery Mildew, Obtained by Cross-Breeding Programs in Spain
Source: Front Plant Sci. 2021 Dec 10;12:674510. doi: 10.3389/fpls.2021.674510 (PMC8703198; doi:10.3389/fpls.2021.674510)
Supplement: Supplementary file 4 [file Table_1.docx]

Supplementary Table 1.- Genetic profile of grapevine material for 9 identificatory SSRs. Alleles expressed in base pairs (bp).

| **Vine material** | **vmc2h10** | **vmc1a12** | **vmc8g6** | **vvmd27** | **vvmd28** | **vvmd5** | **vmc5e9** | **vviv67** | **vvin16** |
| --- | --- | --- | --- | --- | --- | --- | --- | --- | --- |
| Monastrell | 107 116 | 120 138 | 139 173 | 177 187 | 242 256 | 223 237 | 215 228 | 357 364 | 153 159 |
| Regent | 107 112 | 122 122 | 139 159 | 183 187 | 232 256 | 223 235 | 196 211 | 338 372 | 149 153 |
| 3_016 | 107 112 | 122 138 | 139 159 | 183 187 | 256 256 | 223 223 | 196 215 | 357 372 | 149 159 |
| 3_025 | 107 107 | 120 122 | 159 173 | 183 187 | 232 242 | 223 235 | 211 228 | 364 372 | 149 159 |
| 3_032 | 112 116 | 122 138 | 139 173 | 177 187 | 232 256 | 235 237 | 211 215 | 357 372 | 149 159 |
| 3_052 | 107 107 | 120 122 | 139 139 | 177 187 | 256 256 | 223 235 | 211 215 | 338 364 | 153 159 |
| 3_058 | 107 116 | 120 122 | 139 139 | 177 187 | 232 242 | 223 237 | 211 215 | 338 357 | 153 153 |
| 3_070 | 107 116 | 122 138 | 159 173 | 177 183 | 242 256 | 235 237 | 211 228 | 338 364 | 153 153 |
| 3_073 | 107 116 | 122 138 | 139 159 | 187 187 | 256 256 | 223 223 | 196 215 | 338 357 | 153 159 |
| 3_082 | 107 107 | 122 138 | 139 139 | 183 187 | 242 256 | 223 223 | 196 228 | 357 372 | 149 153 |
| 3_094 | 107 112 | 122 138 | 139 159 | 183 187 | 232 242 | 223 237 | 196 228 | 338 357 | 153 159 |
| 4_001 | 107 112 | 122 138 | 139 159 | 177 183 | 256 256 | 223 237 | 196 228 | 338 364 | 153 153 |
| 4_005 | 107 116 | 120 122 | 139 159 | 177 183 | 232 242 | 223 237 | 211 215 | 338 364 | 153 159 |
| 4_011 | 107 107 | 122 138 | 139 173 | 183 187 | 256 256 | 235 237 | 211 215 | 338 364 | 153 159 |
| 4_032 | 107 116 | 122 138 | 159 173 | 183 187 | 232 242 | 223 235 | 196 215 | 364 372 | 149 159 |
| 4_037 | 107 116 | 122 138 | 139 159 | 187 187 | 256 256 | 223 223 | 196 228 | 338 357 | 153 159 |
| 4_063 | 107 116 | 122 138 | 139 159 | 177 187 | 232 242 | 235 237 | 211 215 | 357 372 | 153 159 |
| 4_082 | 107 112 | 120 122 | 159 173 | 177 183 | 232 256 | 235 237 | 211 228 | 364 372 | 153 159 |
| 4_124 | 112 116 | 120 122 | 139 173 | 183 187 | 232 256 | 223 223 | 211 228 | 364 372 | 149 159 |
| 4_136 | 107 112 | 120 122 | 139 173 | 177 187 | 256 256 | 223 223 | 211 228 | 338 357 | 153 153 |
| 5_022 | 107 107 | 120 122 | 159 173 | 187 187 | 232 256 | 235 237 | 211 228 | 338 357 | 149 159 |
| 5_033 | 107 112 | 120 122 | 159 173 | 187 187 | 232 256 | 235 237 | 211 228 | 338 357 | 149 153 |
| 5_060 | 107 107 | 120 122 | 139 173 | 183 187 | 242 256 | 223 237 | 196 215 | 338 357 | 149 153 |
| 5_078 | 107 116 | 122 138 | 159 173 | 183 187 | 232 256 | 223 237 | 196 228 | 338 357 | 153 153 |
| 5_107 | 107 107 | 122 138 | 139 159 | 183 187 | 242 256 | 223 235 | 211 215 | 338 364 | 149 159 |
| 6_018 | 112 116 | 122 138 | 139 139 | 177 183 | 242 256 | 223 237 | 211 215 | 338 357 | 149 159 |
| 6_025 | 112 116 | 120 122 | 139 159 | 177 187 | 256 256 | 223 237 | 211 228 | 338 364 | 149 159 |
| 6_046 | 107 107 | 122 138 | 139 139 | 177 187 | 256 256 | 223 223 | 211 228 | 338 357 | 153 153 |
| 6_080 | 107 107 | 120 122 | 139 139 | 177 187 | 256 256 | 235 237 | 211 228 | 338 357 | 149 159 |
| 6_125 | 107 116 | 122 138 | 139 159 | 183 187 | 242 256 | 235 237 | 211 228 | 338 357 | 149 159 |
