# Supplementary material for: Evaluation and Pre-selection of New Grapevine Genotypes Resistant to Downy and Powdery Mildew, Obtained by Cross-Breeding Programs in Spain
Source: Front Plant Sci. 2021 Dec 10;12:674510. doi: 10.3389/fpls.2021.674510 (PMC8703198; doi:10.3389/fpls.2021.674510)
Supplement: Supplementary file 5 [file Table_2.docx]

Supplementary Table 2.- Sequences of the resistance-associated SSRs for *Rpv3* and *Ren3*.

|  | Markers associated | Marker sequences | |  |
| --- | --- | --- | --- | --- |
| Loci |  | Forward and Reverse primers | | References |
| *Rpv3* | UDV305 | TGGTGCAATGGTCATAATTT | GAGGAAAAGAGAAAGCAAAGA | Bellin et al., 2009; Di Gaspero et al. 2012; Zini et al., 2019 |
|  | UDV737 | TTTGCATGCGATACCTGAAG | TCCTGCAGCTGTTGACGATA | Di Gaspero et al. 2012; Vezzulli et al., 2019; Zini et al., 2019 |
|  | UDV108 | TGTAGGGTTCCAAAGTTCAGG | CCTTTTTATATGTGGTGGAGCA | Di Gaspero et al. 2005; Van Heerden et al., 2014 |
|  | GF18-8 | GACAATAGCGAGAGAGAATGGG | AGTTGGCTAAAACCCTAGAGGC | Zyprian et al. 2016 |
| *Ren3* | GF15-42 | CTCCTAAAATGGACGCAACC | TGCTTCAGGCATTTGAAATCT | Zendler et al. 2017; Zini et al., 2019 |
|  | GF15-28 | TGCACACAATCACAGAGAGAGA | TGCGGTTAATTTTGACTCCTTC | Zyprian et al. 2016 |
|  | GF15-30 | TCACAGTATGCAGTAACCTGGC | AAAGGGAAAATGAGCAGTTGAG | Zyprian et al. 2016 |
|  | VCh15CenGen06 | TGGTCAATGATCTCCCCATT | TTCCAATCAAGGTCATGCAA | Van Heerden et al., 2014; Zendler et al. 2017 |
