# Supplementary material for: Evaluation and Pre-selection of New Grapevine Genotypes Resistant to Downy and Powdery Mildew, Obtained by Cross-Breeding Programs in Spain
Source: Front Plant Sci. 2021 Dec 10;12:674510. doi: 10.3389/fpls.2021.674510 (PMC8703198; doi:10.3389/fpls.2021.674510)
Supplement: Supplementary file 6 [file Table_3.docx]

Supplementary Table 3.- Possession of SSR alleles marking resistance to downy mildew and powdery mildew. Alleles expressed in base pairs (bp).

|  | *Rpv3* (downy mildew) | | | |  | *Ren3* (powdery mildew) | | | |  |  |
| --- | --- | --- | --- | --- | --- | --- | --- | --- | --- | --- | --- |
| **Vine material** | **UDV305_299pb** | **UDV737_279pb** | **UDV108 _238pb** | **GF18-8_392pb** |  | **GF15-42_199pb** | **GF15-28_341pb** | **GF15-30_446pb** | **VCh15CenGen06_283pb** |  | **Genotype R** |
| Monastrell | null 327 | 285 295 | 241 245 | 381 385 |  | 181 185 | 360 366 | 414 434 | 273 null |  | Susceptible |
| Regent | null **299** | **279** 295 | 216 **238** | 387 **392** |  | null **199** | **341** 374 | **446** 462 | 273 **283** |  | DM_PM |
| 3_016 | **299** 327 | **279** 295 | **238** 245 | 385 **392** |  | null 181 | 360 374 | 414 462 | 273 273* |  | DM |
| 3_025 | **299** 327 | **279** 295 | **238** 245 | 385 **392** |  | null 185 | 366 374 | 434 462 | **283** null |  | DM |
| 3_032 | **299** 327 | **279** 295 | **238** 245 | 385 **392** |  | null 181 | 360 374 | 414 462 | 273 273* |  | DM |
| 3_052 | null null | 285 295 | 216 241 | 381 387 |  | 181 **199** | **341** 366 | 434 **446** | **283** null |  | PM |
| 3_058 | null null | 285 295 | 216 241 | 381 387 |  | 185 **199** | **341** 360 | 414 **446** | 273 **283** |  | PM |
| 3_070 | null null | 285 295 | 216 241 | 381 387 |  | 181 **199** | **341** 366 | 434  **446** | **283** null |  | PM |
| 3_073 | null 327 | 295 295 | 216 245 | 385 387 |  | 185 **199** | **341** 360 | 414 **446** | 273 **283** |  | PM |
| 3_082 | null **299** | **279** 285 | **238** 241 | 381 **392** |  | null 181 | 360 374 | 414 462 | 273 273* |  | DM |
| 3_094 | null 327 | 295 295 | 216 245 | 385 387 |  | 181 **199** | **341** 360 | 414 **446** | 273 **283** |  | PM |
| 4_001 | null null | 285 295 | 216 241 | 381 387 |  | 181 **199** | **341** 366 | 434 **446** | **283** null |  | PM |
| 4_005 | null 327 | 295 295 | 216 245 | 385 387 |  | 181 **199** | **341** 366 | 434 **446** | 273 **283** |  | PM |
| 4_011 | null 327 | 295 295 | 216 245 | 385 387 |  | 181 **199** | **341** 366 | 434  **446** | **283** null |  | PM |
| 4_032 | **299** 327 | **279** 295 | **238** 241 | 385 **392** |  | null 181 | 366 374 | 434 462 | 273 273* |  | DM |
| 4_037 | null null | 285 295 | 216 241 | 381 387 |  | 181 **199** | **341** 360 | 414  **446** | 273 **283** |  | PM |
| 4_063 | null **299** | **279** 285 | **238** 241 | 381 **392** |  | null 185 | 360 374 | 434 **446** | 273 273* |  | DM |
| 4_082 | **299** 327 | **279** 295 | **238** 245 | 385 **392** |  | null 181 | 366 374 | 434 462 | 273 273* |  | DM |
| 4_124 | **299** 327 | **279** 295 | **238** 245 | 385 **392** |  | null 185 | 366 374 | 434 **446** | 273 273* |  | DM |
| 4_136 | **299** 327 | **279** 285 | **238** 241 | 381  **392** |  | 181 **199** | **341** 360 | 414 **446** | 273 **283** |  | DM_PM |
| 5_022 | **299** 327 | **279** 295 | **238** 245 | 385 **392** |  | 185 **199** | **341** 360 | 414 **446** | 273 **283** |  | DM_PM |
| 5_033 | null  **299** | **279** 285 | **238** 241 | 381  **392** |  | 185 **199** | **341** 360 | 414 **446** | 273 **283** |  | DM_PM |
| 5_060 | null **299** | **279** 285 | **238** 241 | 381 **392** |  | 185 **199** | 360 374 | 414 462 | 273 **283** |  | DM |
| 5_078 | null null | 285 295 | 216 241 | 381 387 |  | 181 **199** | **341** 360 | 414 **446** | 273 **283** |  | PM |
| 5_107 | **299** 327 | **279** 295 | **238** 245 | 385 **392** |  | 181 **199** | **341** 366 | 434 **446** | **283** null |  | DM_PM |
| 6_018 | **299** 327 | **279** 295 | **238** 245 | 385 **392** |  | 185 **199** | **341** 360 | 414 **446** | 273 **283** |  | DM_PM |
| 6_025 | **299** 327 | **279** 295 | **238** 245 | 385 **392** |  | 181 **199** | **341** 366 | 434 **446** | **283** null |  | DM_PM |
| 6_046 | **299** 327 | **279** 285 | **238** 245 | 385 **392** |  | 181 **199** | **341** 360 | 414 **446** | 273 **283** |  | DM_PM |
| 6_080 | **299** 327 | **279** 295 | **238** 245 | 385 **392** |  | 181 **199** | **341** 360 | 414 **446** | 273 **283** |  | DM_PM |
| 6_125 | **299** 327 | **279** 295 | **238** 245 | 385 **392** |  | 181 **199** | **341** 360 | 414 **446** | 273  **283** |  | DM_PM |

Resistance-associated SSR alleles are shown in bold. DM, Downy mildew-resistant with all four resistance-associated alleles present;

PM, Powdery mildew-resistant with all four resistance-associated alleles present. DM_PM*,* resistant to downy and powdery mildew

*273 273 and 273 null could not be distinguished
