# Supplementary material for: Evaluation and Pre-selection of New Grapevine Genotypes Resistant to Downy and Powdery Mildew, Obtained by Cross-Breeding Programs in Spain
Source: Front Plant Sci. 2021 Dec 10;12:674510. doi: 10.3389/fpls.2021.674510 (PMC8703198; doi:10.3389/fpls.2021.674510)
Supplement: Supplementary file 7 [file Table_4.docx]

Supplementary Table 4.- Association between resistance-associated alleles and actual phenotypic resistance to downy mildew and powdery mildew.

|  |  |  | Alleles of DMR | | Significance | |  |  | Alleles of PMR | | Significance | |
| --- | --- | --- | --- | --- | --- | --- | --- | --- | --- | --- | --- | --- |
|  | Level of resistance | N (%) | (+) | (-) | Chi-square | *p* value |  | N (%) | (+) | (-) | Chi-square | *p* value |
| ***OIV descriptor** | OIV 1 | 10 (37.1%) | 1 (10%) | 9 (90%) | 19.57 | <0.001 |  | 0 (0%) | 0 (0%) | 0 (0%) | 10.70 | < 0.025 |
|  | OIV 3 | 1 (3.7%) | 1 (100%) | 0 (0%) |  |  |  | 1 (3.6%) | 0 (0%) | 1 (100%) |  |  |
|  | OIV 5 | 4 (14.8%) | 4 (100%) | 0 (0%) |  |  |  | 3 (10.7%) | 0 (0%) | 3 (100%) |  |  |
|  | OIV 7 | 6 (22.2%) | 6 (100%) | 0 (0%) |  |  |  | 2 (7.1%) | 1 (50%) | 1 (50%) |  |  |
|  | OIV 9 | 6 (22.2%) | 5 (83%) | 1 (17%) |  |  |  | 22 (78.5%) | 18 (81.8%) | 4 (18.2%) |  |  |

Alleles of DMR = SSR alleles associated with resistance to downy mildew (all four alleles present)

Alleles of PMR = SSR alleles associated with resistance to downy mildew (all four alleles present)

N, number of individuals (%, percentage)

(+), four alleles present

(-), not all four alleles present

*OIV descriptor with small modifications that take into account the severity and incidence of the disease.
